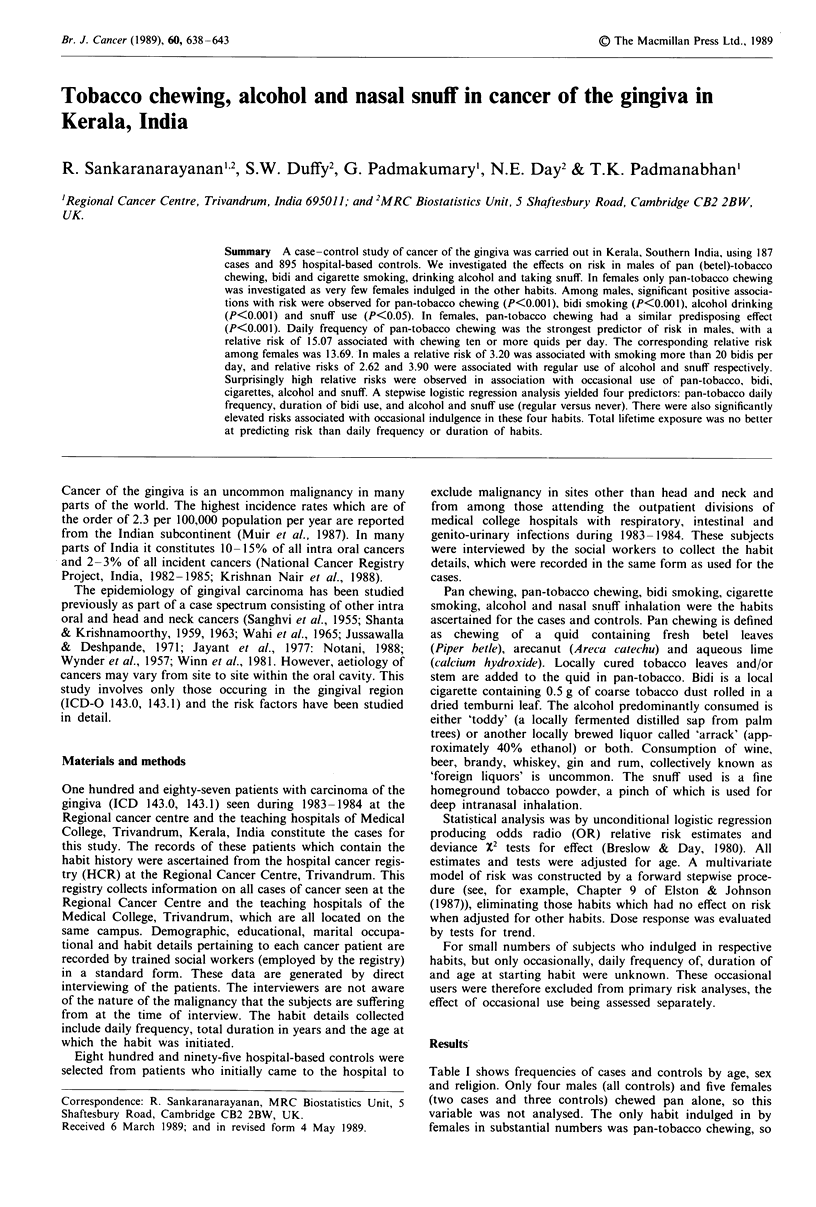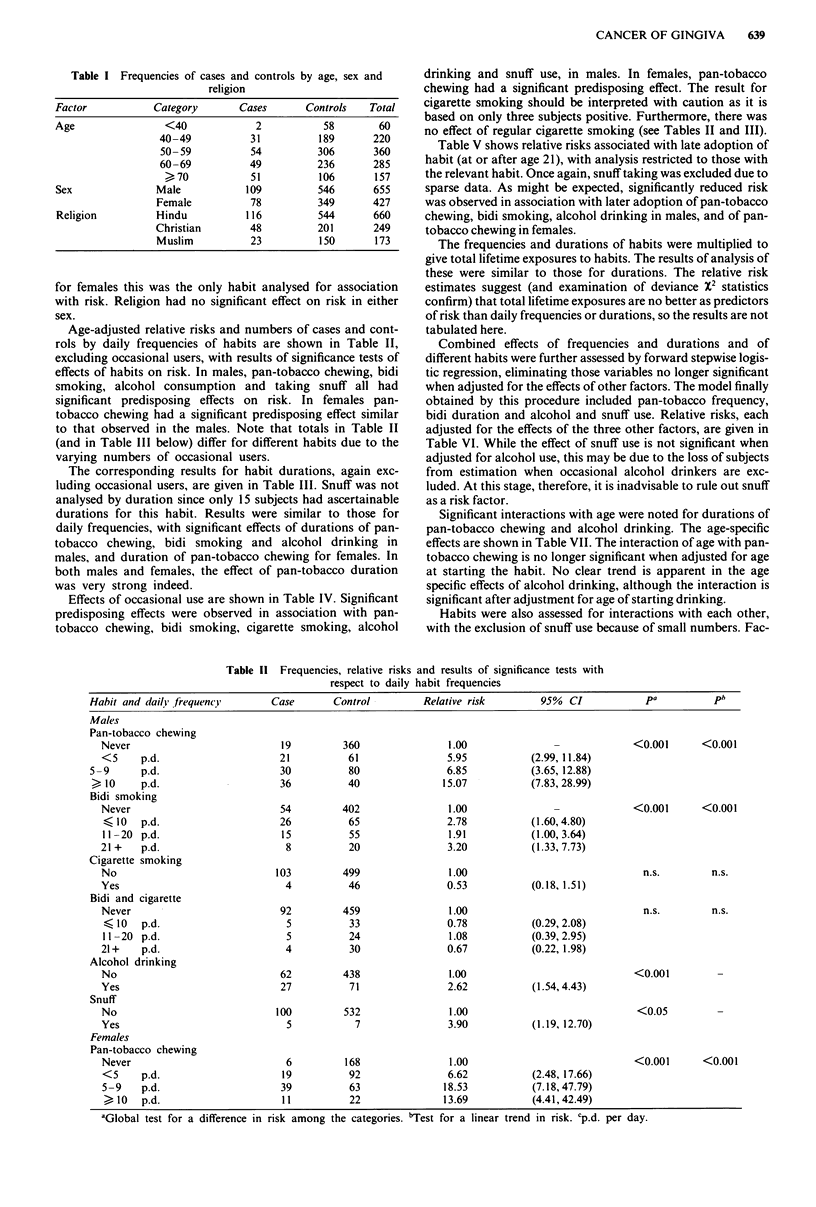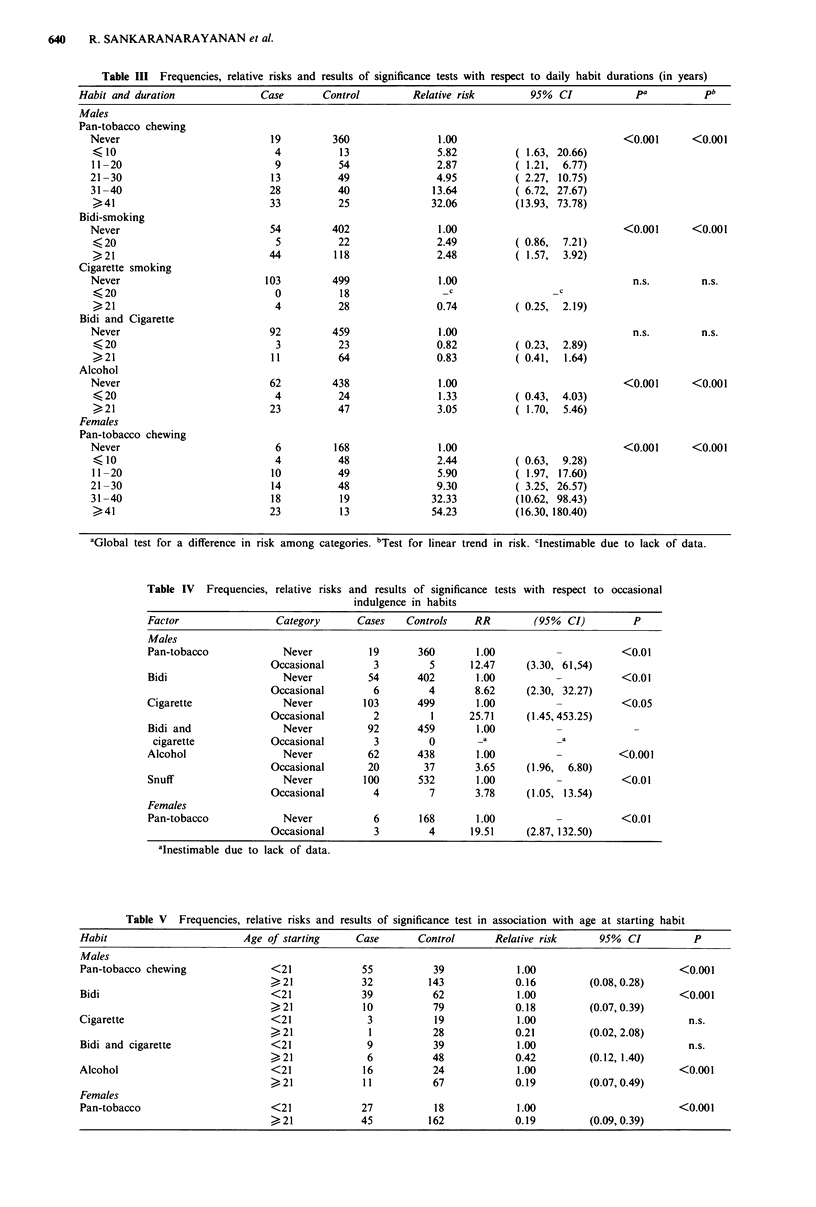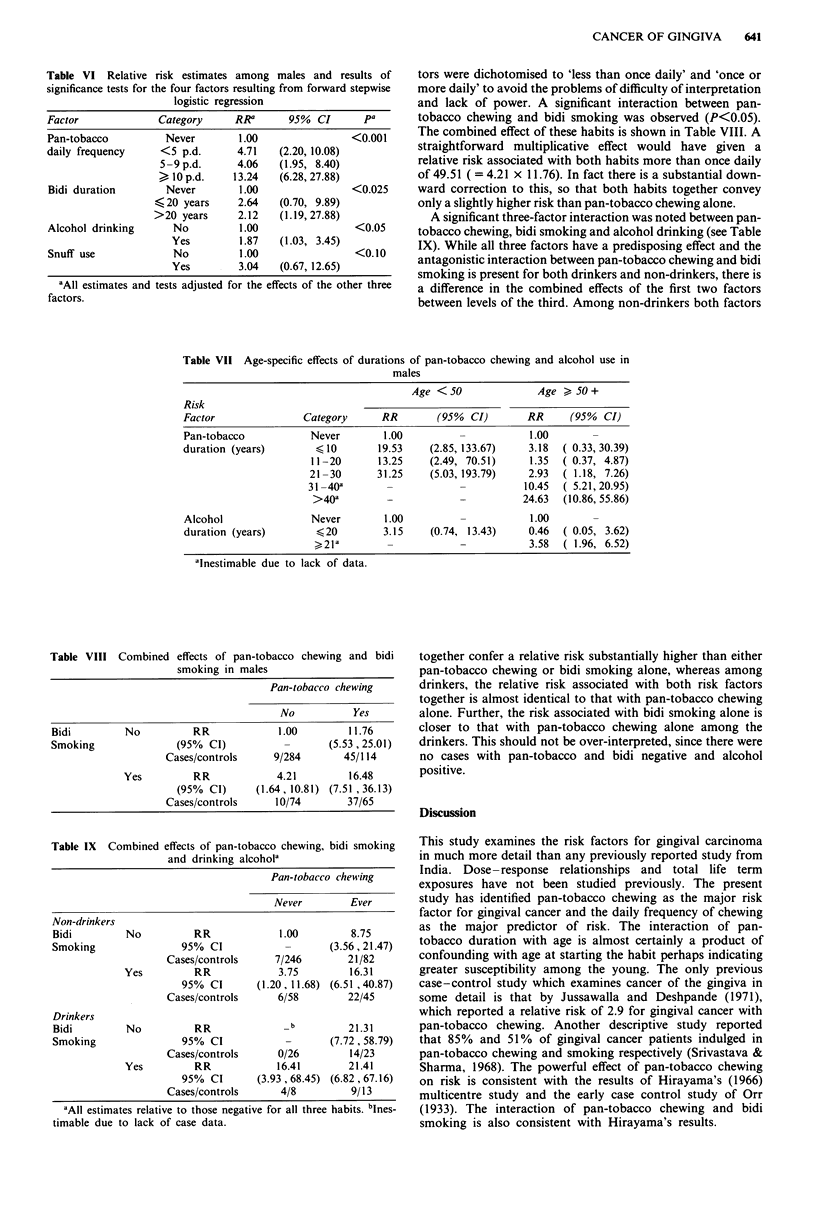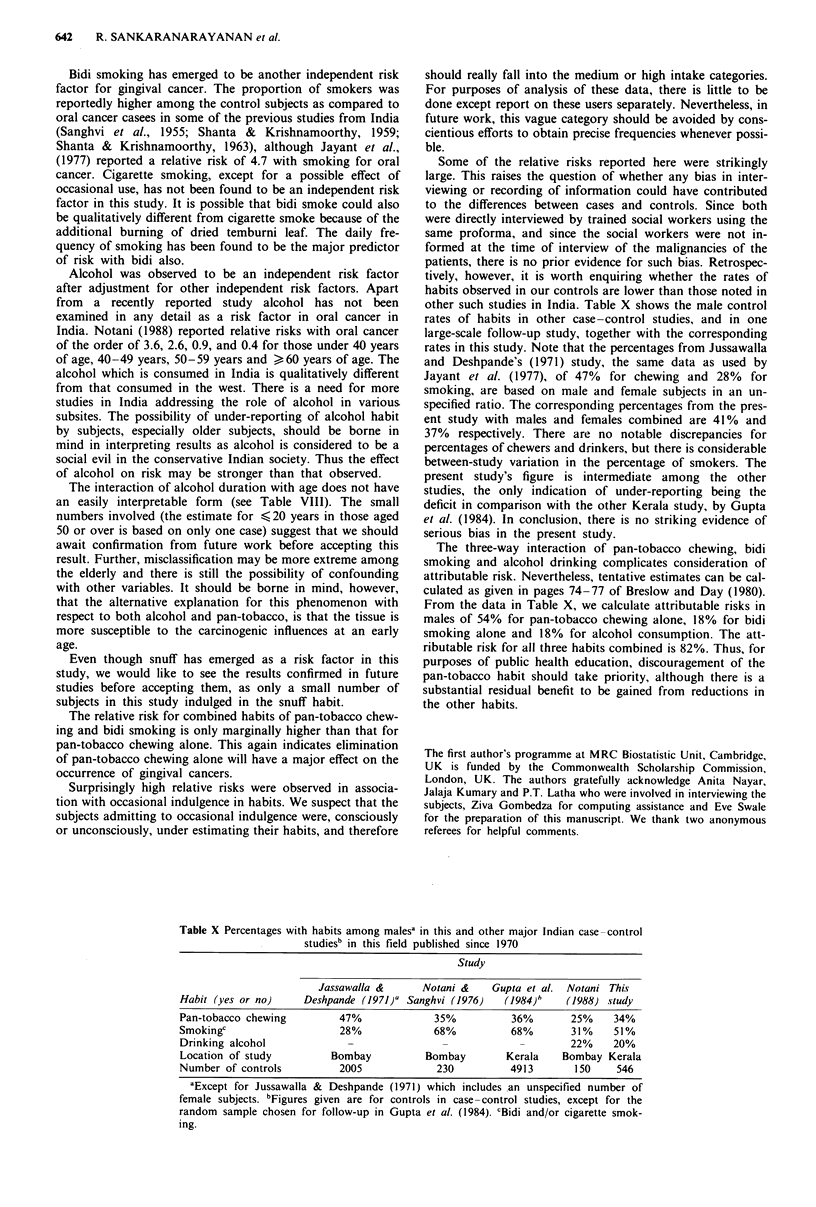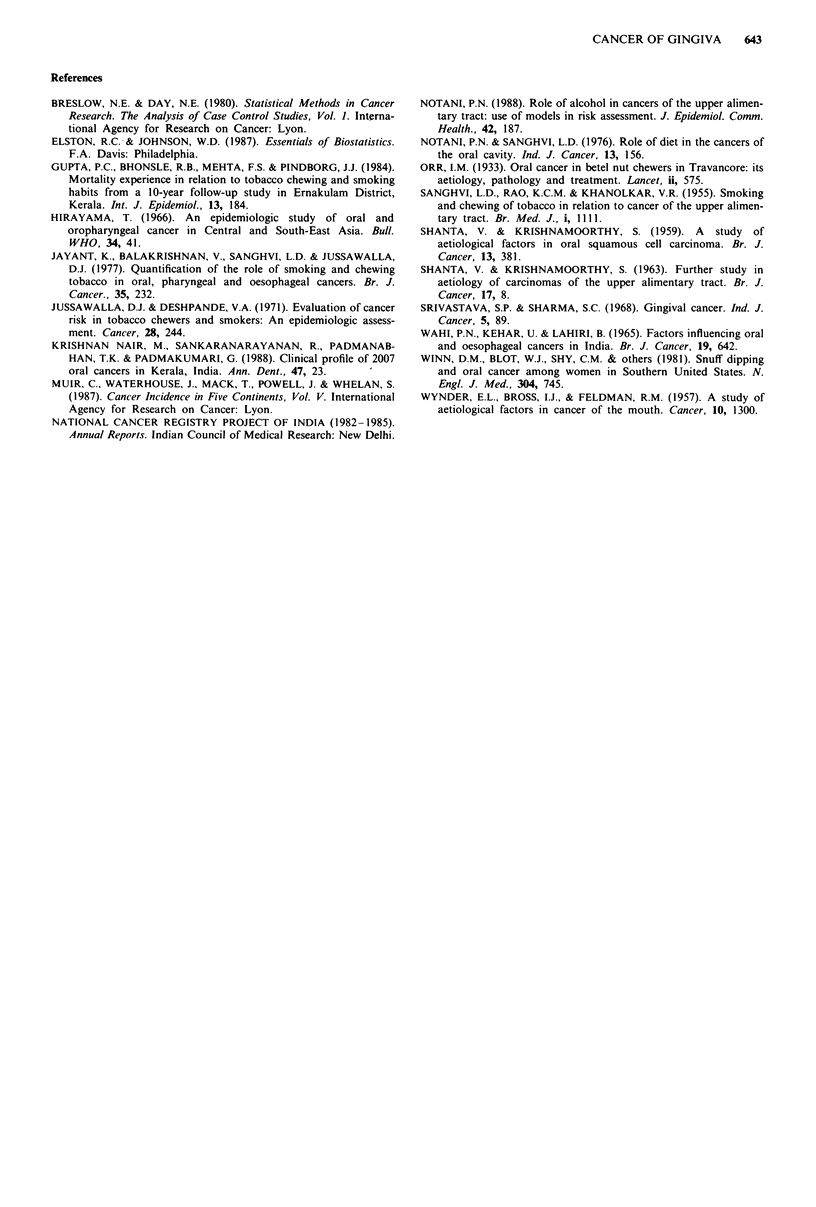# Tobacco chewing, alcohol and nasal snuff in cancer of the gingiva in Kerala, India.

**DOI:** 10.1038/bjc.1989.330

**Published:** 1989-10

**Authors:** R. Sankaranarayanan, S. W. Duffy, G. Padmakumary, N. E. Day, T. K. Padmanabhan

**Affiliations:** Regional Cancer Centre, Trivandrum, India.

## Abstract

A case-control study of cancer of the gingiva was carried out in Kerala, Southern India, using 187 cases and 895 hospital-based controls. We investigated the effects on risk in males of pan (betel)-tobacco chewing, bidi and cigarette smoking, drinking alcohol and taking snuff. In females only pan-tobacco chewing was investigated as very few females indulged in the other habits. Among males, significant positive associations with risk were observed for pan-tobacco chewing (P less than 0.001), bidi smoking (P less than 0.001) alcohol drinking (P less than 0.001) and snuff use (P less than 0.05). In females, pan-tobacco chewing had a similar predisposing effect (P less than 0.001). Daily frequency of pan-tobacco chewing was the strongest predictor of risk in males, with a relative risk of 15.07 associated with chewing ten or more quids per day. The corresponding relative risk among females was 13.69. In males a relative risk of 3.20 was associated with smoking more than 20 bidis per day, and relative risks of 2.62 and 3.90 were associated with regular use of alcohol and snuff respectively. Surprisingly high relative risks were observed in association with occasional use of pan-tobacco, bidi, cigarettes, alcohol and snuff. A stepwise logistic regression analysis yielded four predictors: pan-tobacco daily frequency, duration of bidi use, and alcohol and snuff use (regular versus never). There were also significantly elevated risks associated with occasional indulgence in these four habits. Total lifetime exposure was no better at predicting risk than daily frequency or duration of habits.


Br. J. Cancer (1989), 60, 638–643 © The Macmillan Press Ltd., 1989

### Tobacco chewing, alcohol and nasal snuff in cancer of the gingiva in Kerala, India

R. Sankaranarayanan[1,2], S.W. Duffy[2], G. Padmakumary[1], N.E. Day[2] & T.K. Padmanabhan[1]

[1]Regional Cancer Centre, Trivandrum, India 695011; and [2]MRC Biostatistics Unit, 5 Shaftesbury Road, Cambridge CB2 2BW, UK.

**Summary** A case–control study of cancer of the gingiva was carried out in Kerala, Southern India, using 187 cases and 895 hospital-based controls. We investigated the effects on risk in males of pan (betel)-tobacco chewing, bidi and cigarette smoking, drinking alcohol and taking snuff. In females only pan-tobacco chewing was investigated as very few females indulged in the other habits. Among males, significant positive associations with risk were observed for pan-tobacco chewing ($P<0.001$), bidi smoking ($P<0.001$), alcohol drinking ($P<0.001$) and snuff use ($P<0.05$). In females, pan-tobacco chewing had a similar predisposing effect ($P<0.001$). Daily frequency of pan-tobacco chewing was the strongest predictor of risk in males, with a relative risk of 15.07 associated with chewing ten or more quids per day. The corresponding relative risk among females was 13.69. In males a relative risk of 3.20 was associated with smoking more than 20 bidis per day, and relative risks of 2.62 and 3.90 were associated with regular use of alcohol and snuff respectively. Surprisingly high relative risks were observed in association with occasional use of pan-tobacco, bidi, cigarettes, alcohol and snuff. A stepwise logistic regression analysis yielded four predictors: pan-tobacco daily frequency, duration of bidi use, and alcohol and snuff use (regular versus never). There were also significantly elevated risks associated with occasional indulgence in these four habits. Total lifetime exposure was no better at predicting risk than daily frequency or duration of habits.

Cancer of the gingiva is an uncommon malignancy in many parts of the world. The highest incidence rates which are of the order of 2.3 per 100,000 population per year are reported from the Indian subcontinent (Muir *et al.*, 1987). In many parts of India it constitutes 10–15% of all intra oral cancers and 2–3% of all incident cancers (National Cancer Registry Project, India, 1982–1985; Krishnan Nair *et al.*, 1988).

The epidemiology of gingival carcinoma has been studied previously as part of a case spectrum consisting of other intra oral and head and neck cancers (Sanghvi *et al.*, 1955; Shanta & Krishnamoorthy, 1959, 1963; Wahi *et al.*, 1965; Jussawalla & Deshpande, 1971; Jayant *et al.*, 1977: Notani, 1988; Wynder *et al.*, 1957; Winn *et al.*, 1981. However, aetiology of cancers may vary from site to site within the oral cavity. This study involves only those occuring in the gingival region (ICD-O 143.0, 143.1) and the risk factors have been studied in detail.

### Materials and methods

One hundred and eighty-seven patients with carcinoma of the gingiva (ICD 143.0, 143.1) seen during 1983–1984 at the Regional cancer centre and the teaching hospitals of Medical College, Trivandrum, Kerala, India constitute the cases for this study. The records of these patients which contain the habit history were ascertained from the hospital cancer registry (HCR) at the Regional Cancer Centre, Trivandrum. This registry collects information on all cases of cancer seen at the Regional Cancer Centre and the teaching hospitals of the Medical College, Trivandrum, which are all located on the same campus. Demographic, educational, marital occupational and habit details pertaining to each cancer patient are recorded by trained social workers (employed by the registry) in a standard form. These data are generated by direct interviewing of the patients. The interviewers are not aware of the nature of the malignancy that the subjects are suffering from at the time of interview. The habit details collected include daily frequency, total duration in years and the age at which the habit was initiated.

Eight hundred and ninety-five hospital-based controls were selected from patients who initially came to the hospital to exclude malignancy in sites other than head and neck and from among those attending the outpatient divisions of medical college hospitals with respiratory, intestinal and genito-urinary infections during 1983–1984. These subjects were interviewed by the social workers to collect the habit details, which were recorded in the same form as used for the cases.

Pan chewing, pan-tobacco chewing, bidi smoking, cigarette smoking, alcohol and nasal snuff inhalation were the habits ascertained for the cases and controls. Pan chewing is defined as chewing of a quid containing fresh betel leaves (*Piper betle*), arecanut (*Areca catechu*) and aqueous lime (*calcium hydroxide*). Locally cured tobacco leaves and/or stem are added to the quid in pan-tobacco. Bidi is a local cigarette containing 0.5 g of coarse tobacco dust rolled in a dried temburni leaf. The alcohol predominantly consumed is either 'toddy' (a locally fermented distilled sap from palm trees) or another locally brewed liquor called 'arrack' (approximately 40% ethanol) or both. Consumption of wine, beer, brandy, whiskey, gin and rum, collectively known as 'foreign liquors' is uncommon. The snuff used is a fine homeground tobacco powder, a pinch of which is used for deep intranasal inhalation.

Statistical analysis was by unconditional logistic regression producing odds radio (OR) relative risk estimates and deviance $\chi^2$ tests for effect (Breslow & Day, 1980). All estimates and tests were adjusted for age. A multivariate model of risk was constructed by a forward stepwise procedure (see, for example, Chapter 9 of Elston & Johnson (1987)), eliminating those habits which had no effect on risk when adjusted for other habits. Dose response was evaluated by tests for trend.

For small numbers of subjects who indulged in respective habits, but only occasionally, daily frequency of, duration of and age at starting habit were unknown. These occasional users were therefore excluded from primary risk analyses, the effect of occasional use being assessed separately.

### Results

Table I shows frequencies of cases and controls by age, sex and religion. Only four males (all controls) and five females (two cases and three controls) chewed pan alone, so this variable was not analysed. The only habit indulged in by females in substantial numbers was pan-tobacco chewing, so

Correspondence: R. Sankaranarayanan, MRC Biostatistics Unit, 5 Shaftesbury Road, Cambridge CB2 2BW, UK.
Received 6 March 1989; and in revised form 4 May 1989.

**Table I** Frequencies of cases and controls by age, sex and religion

| *Factor* | *Category* | *Cases* | *Controls* | *Total* |
|---|---|---|---|---|
| Age | <40 | 2 | 58 | 60 |
| | 40–49 | 31 | 189 | 220 |
| | 50–59 | 54 | 306 | 360 |
| | 60–69 | 49 | 236 | 285 |
| | ⩾70 | 51 | 106 | 157 |
| Sex | Male | 109 | 546 | 655 |
| | Female | 78 | 349 | 427 |
| Religion | Hindu | 116 | 544 | 660 |
| | Christian | 48 | 201 | 249 |
| | Muslim | 23 | 150 | 173 |

for females this was the only habit analysed for association with risk. Religion had no significant effect on risk in either sex.

Age-adjusted relative risks and numbers of cases and controls by daily frequencies of habits are shown in Table II, excluding occasional users, with results of significance tests of effects of habits on risk. In males, pan-tobacco chewing, bidi smoking, alcohol consumption and taking snuff all had significant predisposing effects on risk. In females pan-tobacco chewing had a significant predisposing effect similar to that observed in the males. Note that totals in Table II (and in Table III below) differ for different habits due to the varying numbers of occasional users.

The corresponding results for habit durations, again excluding occasional users, are given in Table III. Snuff was not analysed by duration since only 15 subjects had ascertainable durations for this habit. Results were similar to those for daily frequencies, with significant effects of durations of pan-tobacco chewing, bidi smoking and alcohol drinking in males, and duration of pan-tobacco chewing for females. In both males and females, the effect of pan-tobacco duration was very strong indeed.

Effects of occasional use are shown in Table IV. Significant predisposing effects were observed in association with pan-tobacco chewing, bidi smoking, cigarette smoking, alcohol drinking and snuff use, in males. In females, pan-tobacco chewing had a significant predisposing effect. The result for cigarette smoking should be interpreted with caution as it is based on only three subjects positive. Furthermore, there was no effect of regular cigarette smoking (see Tables II and III).

Table V shows relative risks associated with late adoption of habit (at or after age 21), with analysis restricted to those with the relevant habit. Once again, snuff taking was excluded due to sparse data. As might be expected, significantly reduced risk was observed in association with later adoption of pan-tobacco chewing, bidi smoking, alcohol drinking in males, and of pan-tobacco chewing in females.

The frequencies and durations of habits were multiplied to give total lifetime exposures to habits. The results of analysis of these were similar to those for durations. The relative risk estimates suggest (and examination of deviance $\chi^2$ statistics confirm) that total lifetime exposures are no better as predictors of risk than daily frequencies or durations, so the results are not tabulated here.

Combined effects of frequencies and durations and of different habits were further assessed by forward stepwise logistic regression, eliminating those variables no longer significant when adjusted for the effects of other factors. The model finally obtained by this procedure included pan-tobacco frequency, bidi duration and alcohol and snuff use. Relative risks, each adjusted for the effects of the three other factors, are given in Table VI. While the effect of snuff use is not significant when adjusted for alcohol use, this may be due to the loss of subjects from estimation when occasional alcohol drinkers are excluded. At this stage, therefore, it is inadvisable to rule out snuff as a risk factor.

Significant interactions with age were noted for durations of pan-tobacco chewing and alcohol drinking. The age-specific effects are shown in Table VII. The interaction of age with pan-tobacco chewing is no longer significant when adjusted for age at starting the habit. No clear trend is apparent in the age specific effects of alcohol drinking, although the interaction is significant after adjustment for age of starting drinking.

Habits were also assessed for interactions with each other, with the exclusion of snuff use because of small numbers. Fac-

**Table II** Frequencies, relative risks and results of significance tests with respect to daily habit frequencies

| *Habit and daily frequency* | *Case* | *Control* | *Relative risk* | *95% CI* | $P^a$ | $P^b$ |
|---|---|---|---|---|---|---|
| *Males* | | | | | | |
| Pan-tobacco chewing | | | | | | |
| Never | 19 | 360 | 1.00 | – | <0.001 | <0.001 |
| <5 p.d. | 21 | 61 | 5.95 | (2.99, 11.84) | | |
| 5–9 p.d. | 30 | 80 | 6.85 | (3.65, 12.88) | | |
| ⩾10 p.d. | 36 | 40 | 15.07 | (7.83, 28.99) | | |
| Bidi smoking | | | | | | |
| Never | 54 | 402 | 1.00 | – | <0.001 | <0.001 |
| ⩽10 p.d. | 26 | 65 | 2.78 | (1.60, 4.80) | | |
| 11–20 p.d. | 15 | 55 | 1.91 | (1.00, 3.64) | | |
| 21+ p.d. | 8 | 20 | 3.20 | (1.33, 7.73) | | |
| Cigarette smoking | | | | | | |
| No | 103 | 499 | 1.00 | | n.s. | n.s. |
| Yes | 4 | 46 | 0.53 | (0.18, 1.51) | | |
| Bidi and cigarette | | | | | | |
| Never | 92 | 459 | 1.00 | | n.s. | n.s. |
| ⩽10 p.d. | 5 | 33 | 0.78 | (0.29, 2.08) | | |
| 11–20 p.d. | 5 | 24 | 1.08 | (0.39, 2.95) | | |
| 21+ p.d. | 4 | 30 | 0.67 | (0.22, 1.98) | | |
| Alcohol drinking | | | | | | |
| No | 62 | 438 | 1.00 | | <0.001 | – |
| Yes | 27 | 71 | 2.62 | (1.54, 4.43) | | |
| Snuff | | | | | | |
| No | 100 | 532 | 1.00 | | <0.05 | – |
| Yes | 5 | 7 | 3.90 | (1.19, 12.70) | | |
| *Females* | | | | | | |
| Pan-tobacco chewing | | | | | | |
| Never | 6 | 168 | 1.00 | | <0.001 | <0.001 |
| <5 p.d. | 19 | 92 | 6.62 | (2.48, 17.66) | | |
| 5–9 p.d. | 39 | 63 | 18.53 | (7.18, 47.79) | | |
| ⩾10 p.d. | 11 | 22 | 13.69 | (4.41, 42.49) | | |

[a]Global test for a difference in risk among the categories. [b]Test for a linear trend in risk. [c]p.d. per day.

**Table III** Frequencies, relative risks and results of significance tests with respect to daily habit durations (in years)

| *Habit and duration* | *Case* | *Control* | *Relative risk* | *95% CI* | $P^a$ | $P^b$ |
|---|---|---|---|---|---|---|
| *Males* | | | | | | |
| Pan-tobacco chewing | | | | | | |
| Never | 19 | 360 | 1.00 | | <0.001 | <0.001 |
| ⩽10 | 4 | 13 | 5.82 | ( 1.63, 20.66) | | |
| 11-20 | 9 | 54 | 2.87 | ( 1.21, 6.77) | | |
| 21-30 | 13 | 49 | 4.95 | ( 2.27, 10.75) | | |
| 31-40 | 28 | 40 | 13.64 | ( 6.72, 27.67) | | |
| ⩾41 | 33 | 25 | 32.06 | (13.93, 73.78) | | |
| Bidi-smoking | | | | | | |
| Never | 54 | 402 | 1.00 | | <0.001 | <0.001 |
| ⩽20 | 5 | 22 | 2.49 | ( 0.86, 7.21) | | |
| ⩾21 | 44 | 118 | 2.48 | ( 1.57, 3.92) | | |
| Cigarette smoking | | | | | | |
| Never | 103 | 499 | 1.00 | | n.s. | n.s. |
| ⩽20 | 0 | 18 | –[c] | –[c] | | |
| ⩾21 | 4 | 28 | 0.74 | ( 0.25, 2.19) | | |
| Bidi and Cigarette | | | | | | |
| Never | 92 | 459 | 1.00 | | n.s. | n.s. |
| ⩽20 | 3 | 23 | 0.82 | ( 0.23, 2.89) | | |
| ⩾21 | 11 | 64 | 0.83 | ( 0.41, 1.64) | | |
| Alcohol | | | | | | |
| Never | 62 | 438 | 1.00 | | <0.001 | <0.001 |
| ⩽20 | 4 | 24 | 1.33 | ( 0.43, 4.03) | | |
| ⩾21 | 23 | 47 | 3.05 | ( 1.70, 5.46) | | |
| *Females* | | | | | | |
| Pan-tobacco chewing | | | | | | |
| Never | 6 | 168 | 1.00 | | <0.001 | <0.001 |
| ⩽10 | 4 | 48 | 2.44 | ( 0.63, 9.28) | | |
| 11-20 | 10 | 49 | 5.90 | ( 1.97, 17.60) | | |
| 21-30 | 14 | 48 | 9.30 | ( 3.25, 26.57) | | |
| 31-40 | 18 | 19 | 32.33 | (10.62, 98.43) | | |
| ⩾41 | 23 | 13 | 54.23 | (16.30, 180.40) | | |

[a]Global test for a difference in risk among categories. [b]Test for linear trend in risk. [c]Inestimable due to lack of data.

**Table IV** Frequencies, relative risks and results of significance tests with respect to occasional indulgence in habits

| *Factor* | *Category* | *Cases* | *Controls* | *RR* | *(95% CI)* | *P* |
|---|---|---|---|---|---|---|
| *Males* | | | | | | |
| Pan-tobacco | Never | 19 | 360 | 1.00 | - | <0.01 |
| | Occasional | 3 | 5 | 12.47 | (3.30, 61,54) | |
| Bidi | Never | 54 | 402 | 1.00 | - | <0.01 |
| | Occasional | 6 | 4 | 8.62 | (2.30, 32.27) | |
| Cigarette | Never | 103 | 499 | 1.00 | - | <0.05 |
| | Occasional | 2 | 1 | 25.71 | (1.45, 453.25) | |
| Bidi and cigarette | Never | 92 | 459 | 1.00 | - | - |
| | Occasional | 3 | 0 | –[a] | –[a] | |
| Alcohol | Never | 62 | 438 | 1.00 | - | <0.001 |
| | Occasional | 20 | 37 | 3.65 | (1.96, 6.80) | |
| Snuff | Never | 100 | 532 | 1.00 | - | <0.01 |
| | Occasional | 4 | 7 | 3.78 | (1.05, 13.54) | |
| *Females* | | | | | | |
| Pan-tobacco | Never | 6 | 168 | 1.00 | - | <0.01 |
| | Occasional | 3 | 4 | 19.51 | (2.87, 132.50) | |

[a]Inestimable due to lack of data.

**Table V** Frequencies, relative risks and results of significance test in association with age at starting habit

| *Habit* | *Age of starting* | *Case* | *Control* | *Relative risk* | *95% CI* | *P* |
|---|---|---|---|---|---|---|
| *Males* | | | | | | |
| Pan-tobacco chewing | <21 | 55 | 39 | 1.00 | | <0.001 |
| | ⩾21 | 32 | 143 | 0.16 | (0.08, 0.28) | |
| Bidi | <21 | 39 | 62 | 1.00 | | <0.001 |
| | ⩾21 | 10 | 79 | 0.18 | (0.07, 0.39) | |
| Cigarette | <21 | 3 | 19 | 1.00 | | n.s. |
| | ⩾21 | 1 | 28 | 0.21 | (0.02, 2.08) | |
| Bidi and cigarette | <21 | 9 | 39 | 1.00 | | n.s. |
| | ⩾21 | 6 | 48 | 0.42 | (0.12, 1.40) | |
| Alcohol | <21 | 16 | 24 | 1.00 | | <0.001 |
| | ⩾21 | 11 | 67 | 0.19 | (0.07, 0.49) | |
| *Females* | | | | | | |
| Pan-tobacco | <21 | 27 | 18 | 1.00 | | <0.001 |
| | ⩾21 | 45 | 162 | 0.19 | (0.09, 0.39) | |

**Table VI** Relative risk estimates among males and results of significance tests for the four factors resulting from forward stepwise logistic regression

| *Factor* | *Category* | *RR*[a] | *95% CI* | *P*[a] |
|---|---|---|---|---|
| Pan-tobacco | Never | 1.00 | | <0.001 |
| daily frequency | <5 p.d. | 4.71 | (2.20, 10.08) | |
| | 5–9 p.d. | 4.06 | (1.95, 8.40) | |
| | ≥10 p.d. | 13.24 | (6.28, 27.88) | |
| Bidi duration | Never | 1.00 | | <0.025 |
| | ≤20 years | 2.64 | (0.70, 9.89) | |
| | >20 years | 2.12 | (1.19, 27.88) | |
| Alcohol drinking | No | 1.00 | | <0.05 |
| | Yes | 1.87 | (1.03, 3.45) | |
| Snuff use | No | 1.00 | | <0.10 |
| | Yes | 3.04 | (0.67, 12.65) | |

[a]All estimates and tests adjusted for the effects of the other three factors.

tors were dichotomised to 'less than once daily' and 'once or more daily' to avoid the problems of difficulty of interpretation and lack of power. A significant interaction between pan-tobacco chewing and bidi smoking was observed ($P<0.05$). The combined effect of these habits is shown in Table VIII. A straightforward multiplicative effect would have given a relative risk associated with both habits more than once daily of 49.51 ( = 4.21 × 11.76). In fact there is a substantial downward correction to this, so that both habits together convey only a slightly higher risk than pan-tobacco chewing alone.

A significant three-factor interaction was noted between pan-tobacco chewing, bidi smoking and alcohol drinking (see Table IX). While all three factors have a predisposing effect and the antagonistic interaction between pan-tobacco chewing and bidi smoking is present for both drinkers and non-drinkers, there is a difference in the combined effects of the first two factors between levels of the third. Among non-drinkers both factors together confer a relative risk substantially higher than either pan-tobacco chewing or bidi smoking alone, whereas among drinkers, the relative risk associated with both risk factors together is almost identical to that with pan-tobacco chewing alone. Further, the risk associated with bidi smoking alone is closer to that with pan-tobacco chewing alone among the drinkers. This should not be over-interpreted, since there were no cases with pan-tobacco and bidi negative and alcohol positive.

**Table VII** Age-specific effects of durations of pan-tobacco chewing and alcohol use in males

| | | *Age* < *50* | | *Age* ≥ *50* + | |
|---|---|---|---|---|---|
| *Risk Factor* | *Category* | *RR* | *(95% CI)* | *RR* | *(95% CI)* |
| Pan-tobacco | Never | 1.00 | – | 1.00 | – |
| duration (years) | ≤10 | 19.53 | (2.85, 133.67) | 3.18 | ( 0.33, 30.39) |
| | 11–20 | 13.25 | (2.49, 70.51) | 1.35 | ( 0.37, 4.87) |
| | 21–30 | 31.25 | (5.03, 193.79) | 2.93 | ( 1.18, 7.26) |
| | 31–40[a] | – | – | 10.45 | ( 5.21, 20.95) |
| | >40[a] | – | – | 24.63 | (10.86, 55.86) |
| Alcohol | Never | 1.00 | – | 1.00 | – |
| duration (years) | ≤20 | 3.15 | (0.74, 13.43) | 0.46 | ( 0.05, 3.62) |
| | ≥21[a] | – | – | 3.58 | ( 1.96, 6.52) |

[a]Inestimable due to lack of data.

**Table VIII** Combined effects of pan-tobacco chewing and bidi smoking in males

| | | | *Pan-tobacco chewing* | |
|---|---|---|---|---|
| | | | *No* | *Yes* |
| Bidi | No | RR | 1.00 | 11.76 |
| Smoking | | (95% CI) | – | (5.53, 25.01) |
| | | Cases/controls | 9/284 | 45/114 |
| | Yes | RR | 4.21 | 16.48 |
| | | (95% CI) | (1.64, 10.81) | (7.51, 36.13) |
| | | Cases/controls | 10/74 | 37/65 |

**Table IX** Combined effects of pan-tobacco chewing, bidi smoking and drinking alcohol[a]

| | | | *Pan-tobacco chewing* | |
|---|---|---|---|---|
| | | | *Never* | *Ever* |
| *Non-drinkers* | | | | |
| Bidi | No | RR | 1.00 | 8.75 |
| Smoking | | 95% CI | – | (3.56, 21.47) |
| | | Cases/controls | 7/246 | 21/82 |
| | Yes | RR | 3.75 | 16.31 |
| | | 95% CI | (1.20, 11.68) | (6.51, 40.87) |
| | | Cases/controls | 6/58 | 22/45 |
| *Drinkers* | | | | |
| Bidi | No | RR | –[b] | 21.31 |
| Smoking | | 95% CI | – | (7.72, 58.79) |
| | | Cases/controls | 0/26 | 14/23 |
| | Yes | RR | 16.41 | 21.41 |
| | | 95% CI | (3.93, 68.45) | (6.82, 67.16) |
| | | Cases/controls | 4/8 | 9/13 |

[a]All estimates relative to those negative for all three habits. [b]Inestimable due to lack of case data.

### Discussion

This study examines the risk factors for gingival carcinoma in much more detail than any previously reported study from India. Dose–response relationships and total life term exposures have not been studied previously. The present study has identified pan-tobacco chewing as the major risk factor for gingival cancer and the daily frequency of chewing as the major predictor of risk. The interaction of pan-tobacco duration with age is almost certainly a product of confounding with age at starting the habit perhaps indicating greater susceptibility among the young. The only previous case–control study which examines cancer of the gingiva in some detail is that by Jussawalla and Deshpande (1971), which reported a relative risk of 2.9 for gingival cancer with pan-tobacco chewing. Another descriptive study reported that 85% and 51% of gingival cancer patients indulged in pan-tobacco chewing and smoking respectively (Srivastava & Sharma, 1968). The powerful effect of pan-tobacco chewing on risk is consistent with the results of Hirayama's (1966) multicentre study and the early case control study of Orr (1933). The interaction of pan-tobacco chewing and bidi smoking is also consistent with Hirayama's results.

Bidi smoking has emerged to be another independent risk factor for gingival cancer. The proportion of smokers was reportedly higher among the control subjects as compared to oral cancer casees in some of the previous studies from India (Sanghvi *et al.*, 1955; Shanta & Krishnamoorthy, 1959; Shanta & Krishnamoorthy, 1963), although Jayant *et al.*, (1977) reported a relative risk of 4.7 with smoking for oral cancer. Cigarette smoking, except for a possible effect of occasional use, has not been found to be an independent risk factor in this study. It is possible that bidi smoke could also be qualitatively different from cigarette smoke because of the additional burning of dried temburni leaf. The daily frequency of smoking has been found to be the major predictor of risk with bidi also.

Alcohol was observed to be an independent risk factor after adjustment for other independent risk factors. Apart from a recently reported study alcohol has not been examined in any detail as a risk factor in oral cancer in India. Notani (1988) reported relative risks with oral cancer of the order of 3.6, 2.6, 0.9, and 0.4 for those under 40 years of age, 40–49 years, 50–59 years and $\geqslant 60$ years of age. The alcohol which is consumed in India is qualitatively different from that consumed in the west. There is a need for more studies in India addressing the role of alcohol in various subsites. The possibility of under-reporting of alcohol habit by subjects, especially older subjects, should be borne in mind in interpreting results as alcohol is considered to be a social evil in the conservative Indian society. Thus the effect of alcohol on risk may be stronger than that observed.

The interaction of alcohol duration with age does not have an easily interpretable form (see Table VIII). The small numbers involved (the estimate for $\leqslant 20$ years in those aged 50 or over is based on only one case) suggest that we should await confirmation from future work before accepting this result. Further, misclassification may be more extreme among the elderly and there is still the possibility of confounding with other variables. It should be borne in mind, however, that the alternative explanation for this phenomenon with respect to both alcohol and pan-tobacco, is that the tissue is more susceptible to the carcinogenic influences at an early age.

Even though snuff has emerged as a risk factor in this study, we would like to see the results confirmed in future studies before accepting them, as only a small number of subjects in this study indulged in the snuff habit.

The relative risk for combined habits of pan-tobacco chewing and bidi smoking is only marginally higher than that for pan-tobacco chewing alone. This again indicates elimination of pan-tobacco chewing alone will have a major effect on the occurrence of gingival cancers.

Surprisingly high relative risks were observed in association with occasional indulgence in habits. We suspect that the subjects admitting to occasional indulgence were, consciously or unconsciously, under estimating their habits, and therefore should really fall into the medium or high intake categories. For purposes of analysis of these data, there is little to be done except report on these users separately. Nevertheless, in future work, this vague category should be avoided by conscientious efforts to obtain precise frequencies whenever possible.

Some of the relative risks reported here were strikingly large. This raises the question of whether any bias in interviewing or recording of information could have contributed to the differences between cases and controls. Since both were directly interviewed by trained social workers using the same proforma, and since the social workers were not informed at the time of interview of the malignancies of the patients, there is no prior evidence for such bias. Retrospectively, however, it is worth enquiring whether the rates of habits observed in our controls are lower than those noted in other such studies in India. Table X shows the male control rates of habits in other case–control studies, and in one large-scale follow-up study, together with the corresponding rates in this study. Note that the percentages from Jussawalla and Deshpande's (1971) study, the same data as used by Jayant *et al.* (1977), of 47% for chewing and 28% for smoking, are based on male and female subjects in an unspecified ratio. The corresponding percentages from the present study with males and females combined are 41% and 37% respectively. There are no notable discrepancies for percentages of chewers and drinkers, but there is considerable between-study variation in the percentage of smokers. The present study's figure is intermediate among the other studies, the only indication of under-reporting being the deficit in comparison with the other Kerala study, by Gupta *et al.* (1984). In conclusion, there is no striking evidence of serious bias in the present study.

The three-way interaction of pan-tobacco chewing, bidi smoking and alcohol drinking complicates consideration of attributable risk. Nevertheless, tentative estimates can be calculated as given in pages 74–77 of Breslow and Day (1980). From the data in Table X, we calculate attributable risks in males of 54% for pan-tobacco chewing alone, 18% for bidi smoking alone and 18% for alcohol consumption. The attributable risk for all three habits combined is 82%. Thus, for purposes of public health education, discouragement of the pan-tobacco habit should take priority, although there is a substantial residual benefit to be gained from reductions in the other habits.

The first author's programme at MRC Biostatistic Unit, Cambridge, UK is funded by the Commonwealth Scholarship Commission, London, UK. The authors gratefully acknowledge Anita Nayar, Jalaja Kumary and P.T. Latha who were involved in interviewing the subjects, Ziva Gombedza for computing assistance and Eve Swale for the preparation of this manuscript. We thank two anonymous referees for helpful comments.

**Table X** Percentages with habits among males[a] in this and other major Indian case–control studies[b] in this field published since 1970

| | Study | | | | |
|---|---|---|---|---|---|
| *Habit (yes or no)* | *Jassawalla & Deshpande (1971)*[a] | *Notani & Sanghvi (1976)* | *Gupta et al. (1984)*[b] | *Notani (1988)* | *This study* |
| Pan-tobacco chewing | 47% | 35% | 36% | 25% | 34% |
| Smoking[c] | 28% | 68% | 68% | 31% | 51% |
| Drinking alcohol | – | – | – | 22% | 20% |
| Location of study | Bombay | Bombay | Kerala | Bombay | Kerala |
| Number of controls | 2005 | 230 | 4913 | 150 | 546 |

[a]Except for Jussawalla & Deshpande (1971) which includes an unspecified number of female subjects. [b]Figures given are for controls in case–control studies, except for the random sample chosen for follow-up in Gupta *et al.* (1984). [c]Bidi and/or cigarette smoking.

### References

BRESLOW, N.E. & DAY, N.E. (1980). *Statistical Methods in Cancer Research. The Analysis of Case Control Studies, Vol. 1.* International Agency for Research on Cancer: Lyon.

ELSTON, R.C. & JOHNSON, W.D. (1987). *Essentials of Biostatistics.* F.A. Davis: Philadelphia.

GUPTA, P.C., BHONSLE, R.B., MEHTA, F.S. & PINDBORG, J.J. (1984). Mortality experience in relation to tobacco chewing and smoking habits from a 10-year follow-up study in Ernakulam District, Kerala. *Int. J. Epidemiol.*, **13**, 184.

HIRAYAMA, T. (1966). An epidemiologic study of oral and oropharyngeal cancer in Central and South-East Asia. *Bull. WHO*, **34**, 41.

JAYANT, K., BALAKRISHNAN, V., SANGHVI, L.D. & JUSSAWALLA, D.J. (1977). Quantification of the role of smoking and chewing tobacco in oral, pharyngeal and oesophageal cancers. *Br. J. Cancer.*, **35**, 232.

JUSSAWALLA, D.J. & DESHPANDE, V.A. (1971). Evaluation of cancer risk in tobacco chewers and smokers: An epidemiologic assessment. *Cancer*, **28**, 244.

KRISHNAN NAIR, M., SANKARANARAYANAN, R., PADMANABHAN, T.K. & PADMAKUMARI, G. (1988). Clinical profile of 2007 oral cancers in Kerala, India. *Ann. Dent.*, **47**, 23.

MUIR, C., WATERHOUSE, J., MACK, T., POWELL, J. & WHELAN, S. (1987). *Cancer Incidence in Five Continents, Vol. V.* International Agency for Research on Cancer: Lyon.

NATIONAL CANCER REGISTRY PROJECT OF INDIA (1982–1985). *Annual Reports.* Indian Council of Medical Research: New Delhi.

NOTANI, P.N. (1988). Role of alcohol in cancers of the upper alimentary tract: use of models in risk assessment. *J. Epidemiol. Comm. Health.*, **42**, 187.

NOTANI, P.N. & SANGHVI, L.D. (1976). Role of diet in the cancers of the oral cavity. *Ind. J. Cancer*, **13**, 156.

ORR, I.M. (1933). Oral cancer in betel nut chewers in Travancore: its aetiology, pathology and treatment. *Lancet*, **ii**, 575.

SANGHVI, L.D., RAO, K.C.M. & KHANOLKAR, V.R. (1955). Smoking and chewing of tobacco in relation to cancer of the upper alimentary tract. *Br. Med. J.*, **i**, 1111.

SHANTA, V. & KRISHNAMOORTHY, S. (1959). A study of aetiological factors in oral squamous cell carcinoma. *Br. J. Cancer*, **13**, 381.

SHANTA, V. & KRISHNAMOORTHY, S. (1963). Further study in aetiology of carcinomas of the upper alimentary tract. *Br. J. Cancer*, **17**, 8.

SRIVASTAVA, S.P. & SHARMA, S.C. (1968). Gingival cancer. *Ind. J. Cancer*, **5**, 89.

WAHI, P.N., KEHAR, U. & LAHIRI, B. (1965). Factors influencing oral and oesophageal cancers in India. *Br. J. Cancer*, **19**, 642.

WINN, D.M., BLOT, W.J., SHY, C.M. & others (1981). Snuff dipping and oral cancer among women in Southern United States. *N. Engl. J. Med.*, **304**, 745.

WYNDER, E.L., BROSS, I.J., & FELDMAN, R.M. (1957). A study of aetiological factors in cancer of the mouth. *Cancer*, **10**, 1300.